# Ten-year trends in intensive care admissions for respiratory infections in the elderly

**DOI:** 10.1186/s13613-018-0430-6

**Published:** 2018-08-15

**Authors:** Lucile Laporte, Coralie Hermetet, Youenn Jouan, Christophe Gaborit, Emmanuelle Rouve, Kimberly M. Shea, Mustapha Si-Tahar, Pierre-François Dequin, Leslie Grammatico-Guillon, Antoine Guillon

**Affiliations:** 1 Université de Tours, Tours, France; 2 Service de Médecine Intensive Réanimation, CHRU de Tours, 2 Bd Tonnellé, 37044 Tours Cedex 9, France; 3 Service d’Information Médicale, d’Epidémiologie et d’Economie de la Santé, CHRU Tours, Tours, France; 4 EE EES, Université de Tours, Tours, France; 5 Centre d’Etude des Pathologies Respiratoires, INSERM U1100, Tours, France; 6 Boston University Schools of Public Health, Boston, MA USA

**Keywords:** Respiratory infection, Intensive care unit, Elderly, Epidemiology

## Abstract

**Background:**

The consequences of the ageing population concerning ICU hospitalisation need to be adequately described. We believe that this discussion should be disease specific. A focus on respiratory infections is of particular interest, because it is strongly associated with old age. Our objective was to assess trends in demographics over a decade among elderly patients admitted to the ICU for acute respiratory infections.

**Methods:**

A cross-sectional study was performed between 2006 and 2015 based on hospital discharge databases in one French region (2.5 million inhabitants). Patients with acute respiratory infection were selected according to the specific ICD-10 diagnosis codes recorded, including acute exacerbation of chronic obstructive pulmonary disease (AECOPD) and community-acquired pneumonia (CAP). We also identified comorbid conditions based on any significant ICD-10 secondary diagnoses adapted from the Charlson and Elixhauser indexes.

**Results:**

A total of 98,381 hospital stays for acute respiratory infection were identified among the 3,856,785 stays over the 10-year period. The number of patients 75 y/o and younger increased 1.6-fold from 2006 to 2015, whereas the numbers of patients aged 85–89 and ≥ 90 y/o increased by 2.5- and 2.1-fold, respectively. Both CAP and AECOPD hospitalisations significantly increased for all age groups over the decade. ICU hospitalisations for respiratory infection increased 2.7-fold from 2006 to 2015 (*p* = 0.0002). The greatest increases in the use of ICU resources were for the 85–89 and ≥ 90 y/o groups, which corresponded to increases of 3.3- and 5.8-fold. Indeed, the proportion of patients hospitalized for respiratory infection in ICU that were elderly clearly grew during the decade: 11.3% were ≥ 85 y/o in 2006 versus 16.4% in 2015 (*p* < 0.0001). This increase in ICU hospitalisation rate of ageing patients was not associated with significant changes in the level of care or ICU mortality except for patients ≥ 90 y/o (for whom ICU mortality dropped from 40.9 to 22.3%, *p* = 0.03).

**Conclusion:**

We observed a substantial increase in acute respiratory infection diagnoses associated with hospitalisation between 2006 and 2015, with a growing demand for critical care services. Both the absolute number and the percentage of elderly patient ICU admissions increased over the last decade, with the greatest increases being observed for patients 85 years and older.

**Electronic supplementary material:**

The online version of this article (10.1186/s13613-018-0430-6) contains supplementary material, which is available to authorized users.

## Background

Current predictions indicate that by 2050, the percentage of the population that will be 80 years old or older will double, representing nearly 10% of the European and 8% of the North American population [1]. Furthermore, female life expectancy is anticipated to break the 90-year barrier in several industrialised countries [2]; the number of centenarians in the world is projected to increase rapidly from approximately 441,000 in 2013 to 3.4 million in 2050 and 20.1 million in 2100 [3]. An important consequence of longer life expectancies is that this growth of the ageing population may result in increasing demand for critical care and affect the composition of the patient population cared for in ICUs. This will likely lead to an increase in the number of new critical care beds, which are particularly resource-consuming. Thus, the consequences of the increasingly ageing population on the public health system need to be adequately described and understood, especially concerning ICU hospitalisation.

Previous reports demonstrate that in Europe, elderly patients comprise 10–20% of all ICU admissions [4–10]. Longitudinal studies further demonstrate that the proportion of patients admitted to the ICU who are elderly is increasing over time [4]. Most studies considering ICU admission policies and their potential benefits have focused on the elderly as a single entity. We believe that the discussion should be disease specific. It is questionable to discuss hospital admission policies for patients based solely on their advanced age without considering the acute disease leading to ICU admission. Indeed, as critical care admissions for a particular diagnosis decline, those due to other diseases may replace them. These shifts in-hospital diagnoses may mask increases in the incidence of specific diseases. A recent retrospective study of a 7-year period (in the Netherlands) demonstrated a crude decrease in ICU and in-hospital mortality (4.6% and 9.7%, respectively) for patients 80 years and older [11]. Interestingly, this decrease was not observed in all the subgroups studied. In particular, the ICU mortality in patients 80 and over who were admitted for a medical reason was constant during the study period, in contrast to the other admission subgroups. From our point of view, a focus on respiratory infections is of particular interest, because these infections are strongly associated with old age. Pneumonia was considered to be “the old man’s friend”, a terminal event leading to the rapid and relatively painless death of the elderly, avoiding “cold gradations of decay so distressing to himself and to his friends”, as described in 1898 by W. Osler [12]. Today, pneumonia is still associated with considerable mortality (10% of in-hospital mortalities according to population-based studies [13]), but it does not systematically sentence the elderly to death. Consequently, pneumonia and more generally acute respiratory infections (ARI) lead to the utilisation of substantial healthcare resources, in particular ICU hospitalisation for advanced monitoring and live support. We hypothesised that the increase in the percentage of ageing patients in the population is likely to inevitably increase hospital stays for ARI and the utilisation of ICU resources. The objective of our study was to describe and assess trends in demographics over a decade among elderly patients admitted to an ICU for ARI.

## Methods

### Study design and data source

We used medical data collected from the French National Hospital Discharge Database [HDD; *Programme de Médicalisation des Systèmes d’Information (*PMSI)] to estimate the incidence of hospitalised ARI in the Centre-Val de Loire (CVL) region of France from 1 January 2006 to 31 December 2015. In France, it is mandatory to report data from all hospital stays at public and private hospitals; all information from reported hospitalisations is stored in the HDD as medical codes [International Classification of Diseases, Tenth Revision (ICD-10)]. All patients are assigned a unique identification number, allowing the same individual to be followed over time. The CVL region of France has 2.5 million inhabitants and is served by one teaching and regional hospital, one regional hospital and 37 local hospitals.

### Study population

**Fig. 1:**
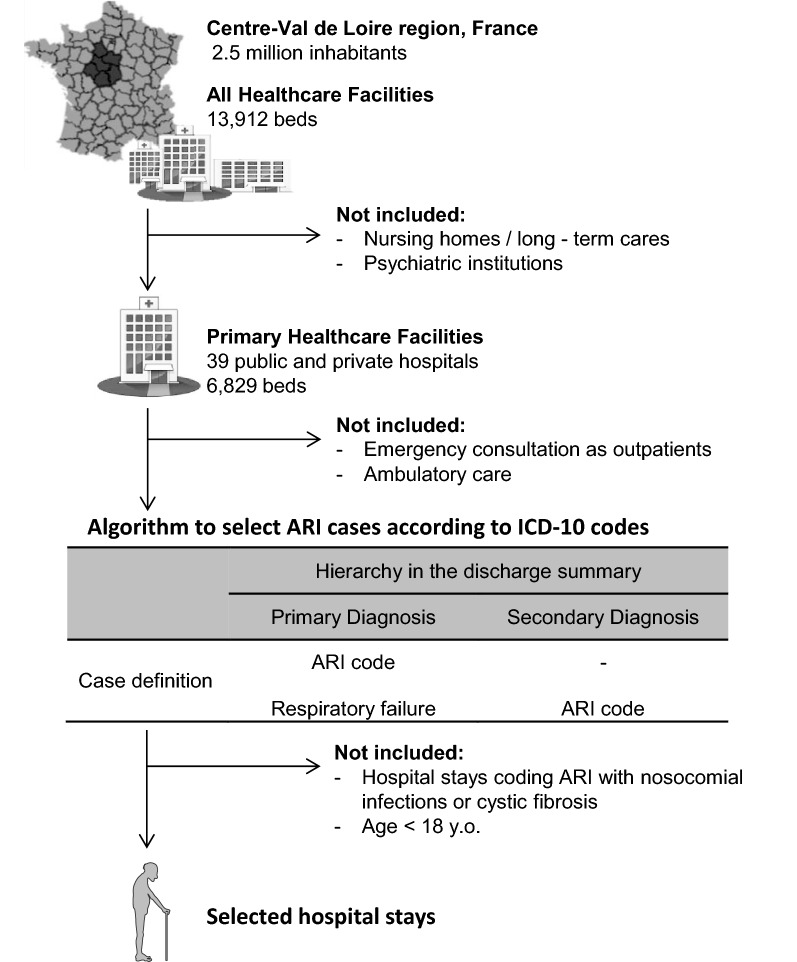
Selection algorithm of acute respiratory infection (ARI) cases according to ICD-10 codes, 2006–2015. ICD-10: International Classification of Diseases, Tenth Revision

The study population comprised all patients aged 18 years or older who were hospitalised at least one time in the CVL region during the study period and who met the ARI case definition (described below). Inpatients with cystic fibrosis or evidence of a nosocomial infection during their hospital stay were not included (Fig. 1).

### ARI case definition

Specific ICD-10 diagnosis codes recorded in patient discharge summaries used to assign ARI status. ARI included diagnosis codes for acute exacerbation of chronic obstructive pulmonary disease (AECOPD) and community-acquired pneumonia (CAP). The codes recorded were: AECOPD (J440, J441), CAP (bacterial CAP: J13, J14, J150–J160, J170; viral CAP: J09, J100, J101, J108, J110, J111, J118, J120–J123, J128, J129; unspecified aetiology of CAP: J168, J172, J173, J178, J180–J182, J188, J189, J690, J851). Hospitalised patients who received at least one of these ICD-10 diagnosis codes as (1) the primary diagnosis in their discharge summary or in the hospital unit where the patient was admitted or (2) the secondary diagnosis with a primary diagnosis of respiratory failure (ICD-10 code J960) were defined as having hospitalised ARI (Fig. 1).

### Other variables

Patient age at the time of hospitalisation was calculated using date of birth and was classified into five categories (< 75, 75–79, 80–84, 85–89 and ≥ 90 years). ICU admission (included intermediate care beds) was ascertained using administrative enrolment data from diagnosis-related groups (DRGs), and death was ascertained using data from discharge administrative records. We identified comorbid conditions based on significant ICD-10 associated with diagnosis adapted and pooled from the Charlson and Elixhauser comorbidity algorithms [14–16]. ICU stay characteristics were recorded over the 10-year period from the HDD; SAPS II scores were available from 2010.

### Statistical analyses

The number and distribution of hospitalised ARI patients were described over time. The in-hospital annual rate of ARI was calculated as the number of hospital admissions attributed to ARI divided by all hospital admissions for any reason during the same study year. The hospital case fatality rate for ARI was calculated as the number of deaths that occurred among hospitalised ARI patients. The rate of ICU admissions for ARI was calculated as the number of ICU admissions attributed to ARI divided by all ARI hospital admissions. To assess heterogeneity in the triage process between regional hospitals (*n* = 2) and local hospitals (*n* = 37), the ICU admission rates were compared. The ICU case fatality rate was calculated as the number of deaths that occurred among ARI ICU patients. The descriptive results were expressed as frequencies (*N*, %). Next, we estimated the annual incidence of hospitalised ARI patients stratified by age as the annual number of hospital admissions attributed to ARI divided by the total annual regional population recorded during the last French census as reported by the National Institute of Statistics and Economic Studies 2013 [17]. The means of incidences by age were expressed as mean ± standard deviation (SD). We used linear regression to estimate the change in ARI hospitalisations over time from 2006 to 2015; correlations were evaluated using the Pearson correlation test, and *p* < 0.05 was considered statistically significant. We used Chi-squared test for linear trend to assess the changes in life support techniques and medicines. Analyses were performed using SAS version 9.1 software for Microsoft Windows (SAS Institute, Cary, NC).

## Results

### Hospitalisations for ARI from 2006 to 2015

**Fig. 2:**
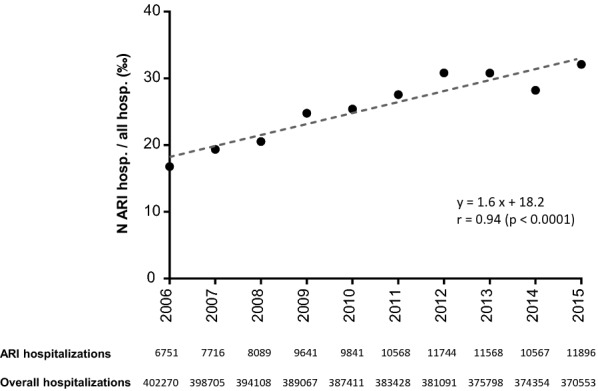
Ten-year trends of in-hospital annual rate of ARI. The in-hospital annual rate of ARI is the number of admissions for ARI divided by overall admissions (all causes of admission) on the same year period. *ARI* acute respiratory infections

We identified 98,381 adult hospital stays attributed to ARI among 3,856,785 total hospital stays in the CVL region from 2006 to 2015. Over time, we observed a marked and consistent increase in the number of annual ARI hospitalisations with a simultaneous decline in the number of all-cause hospitalisations. The number of ARI hospitalisations increased from 6751 (1.7% of all hospitalisations) in 2006 to 11,896 (3.2% of all hospitalisations) in 2015, representing an average annual increase in ARI hospitalisations equal to 5.8% (± 7.9%) over time (*r* = 0.94; *p* < 0.0001). In contrast, the overall number of hospitalisations within this region decreased from 402,270 stays in 2006 to 370,553 in 2015, representing an average annual decrease equal to 0.92% (± 0.37%) (*p* < 0.0001). The in-hospital annual rate of ARI (number of admissions for ARI divided by overall admissions) is represented in Fig. 2.

 The number of ARI hospitalisations increased from 2006 to 2015 in all age groups, but increased proportionally more among patients over 85 years of age (Table 1, Additional file 1: Table S1A).

**Table 1:** Characteristics of hospitalisations for ARI in 2006 and 2015

	2006	2009	2012	2015	*p* value
All-cause hospitalisations	402,270	389,067	381,091	370,553	< 0.0001
ARI hospitalisations	6751	9641	11,744	11,896	< 0.0001
Age					
< 75 y-o	44.4%	44.4%	40.5%	41.0%	< 0.0001
75–79 y-o	14.1%	12.4%	12.8%	11.3%	< 0.0001
80–84 y-o	16.2%	16.0%	16.0%	15.2%	0.002
85–89 y-o	11.6%	16.2%	17.1%	16.5%	< 0.0001
≥ 90 y-o	13.7%	11.0%	13.6%	16.0%	< 0.0001
ARI ICU hospitalisations	740	1239	2135	2036	0.0002
Age					
< 75 y-o	59.7%	61.3%	54.7%	58.2%	< 0.0001
75–79 y-o	14.5%	13.2%	15.0%	12.2%	0.014
80–84 y-o	14.5%	12.7%	14.4%	13.2%	0.167
85–89 y-o	8.3%	9.0%	11.0%	10.2%	0.225
≥ 90 y-o	3.0%	3.8%	4.9%	6.2%	< 0.0001
Sex ratio (M/F)					
< 75 y-o	2.07	1.75	1.95	2.07	0.646
75–79 y-o	1.97	1.81	1.70	2.06	0.677
80–84 y-o	1.49	1.38	1.67	1.55	0.083
85–89 y-o	0.88	0.90	1.04	1.03	0.380
≥ 90 y-o	1.20	0.96	1.0	0.51	0.161
SAPS II (mean ± SD)					
Included age					
< 75 y-o	–	33.6 ± 17.0^a^	34.0 ± 17.3	36.4 ± 17.4	0.083
75–79 y-o	–	38.9 ± 13.3^a^	40.0 ± 16.4	42.6 ± 15.7	0.173
80–84 y-o	–	41.7 ± 13.8^a^	42.3 ± 17.2	44.3 ± 17.9	0.348
85–89 y-o	–	40.8 ± 13.2^a^	41.5 ± 15.0	41.3 ± 14.0	0.665
≥ 90 y-o	–	39.3 ± 12.6^a^	39.9 ± 13.2	40.0 ± 10.7	0.726
Excluded age					
< 75 y-o	–	24.4 ± 16.1^a^	24.4 ± 16.5	26.4 ± 16.7	0.083
75–79 y-o	–	22.9 ± 13.3^a^	24.0 ± 16.4	26.6 ± 15.7	0.173
80–84 y-o	–	23.7 ± 13.8^a^	24.3 ± 17.2	26.3 ± 17.9	0.348
85–89 y-o	–	22.8 ± 13.2^a^	23.5 ± 15.0	23.3 ± 14.0	0.665
≥ 90 y-o	–	21.3 ± 12.6^a^	21.9 ± 13.2	22.0 ± 10.7	0.726
Invasive MV (*n*, %)					
< 75 y-o	172 (38.9)	224 (29.5)	425 (36.4)	440 (37.1)	0.037
75–79 y-o	43 (40.2)	51 (31.3)	77 (24.0)	75 (30.2)	0.007
80–84 y-o	33 (30.8)	49 (31.2)	90 (29.3)	74 (27.6)	0.388
85–89 y-o	18 (29.0)	18 (16.1)	34 (14.5)	31 (15.0)	0.602
≥ 90 y-o	0 (0)	2 (4.3)	12 (11.5)	8 (6.3)	0.141
Non-invasive MV (*n*, %)					
< 75 y-o	92 (20.8)	165 (21.7)	353 (30.2)	447 (37.7)	< 0.0001
75–79 y-o	25 (23.4)	51 (31.3)	100 (31.2)	95 (38.3)	0.038
80–84 y-o	31 (29.0)	43 (27.4)	102 (33.2)	103 (38.4)	0.004
85–89 y-o	19 (30.6)	22 (19.6)	78 (33.2)	56 (27.1)	0.001
≥ 90 y-o	1 (4.5)	3 (6.4)	24 (23.1)	40 (31.5)	< 0.0001
Vasopressor (*n*, %)					
< 75 y-o	101 (22.9)	133 (17.5)	271 (23.2)	308 (26.0)	0.033
75–79 y-o	37 (34.6)	29 (17.8)	57 (17.8)	55 (22.2)	0.049
80–84 y-o	21 (19.6)	24 (15.3)	64 (20.8)	52 (19.4)	0.374
85–89 y-o	11 (17.7)	11 (9.8)	23 (9.8)	24 (11.6)	0.825
≥ 90 y-o	2 (9.1)	1 (2.1)	12 (11.5)	7 (5.5)	0.136
LOS in ICU (day, mean ± SD)					
< 75 y-o	8.3 ± 13.0	7.5 ± 10.0	8.7 ± 12.7	10.6 ± 16.8	0.067
75–79 y–o	11.8 ± 22.9	8.6 ± 13.5	8.3 ± 12.4	9.8 ± 15.1	0.447
80–84 y-o	7.0 ± 10.5	8.6 ± 11.4	8.0 ± 10.1	8.2 ± 9.3	0.595
85–89 y-o	5.2 ± 5.5	5.6 ± 5.1	5.9 ± 6.3	6.2 ± 6.7	0.012
≥ 90 y-o	3.7 ± 3.4	3.8 ± 3.3	5.1 ± 4.9	6.0 ± 6.7	0.003

Four representative years are presented in the table. The statistical analyses were performed on the complete 10-year trends

*y-o* years old, *M* male, *F* female, *ICU* intensive care unit, *ARI* acute respiratory infection, *MV* mechanical ventilation, *LOS* length of stay

^a^SAPS score from 2010, not available on hospital discharge database before

**Fig. 3:**
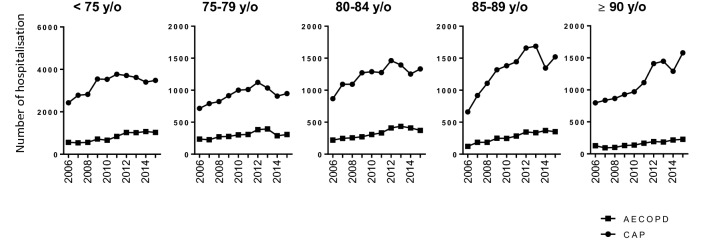
Ten-year trends in hospitalisation for CAP and AECOPD by age class. All increases in CAP and AECOPD hospitalisations over time from 2006 to 2015 were statistically significant. *AECOPD* acute exacerbation of chronic obstructive pulmonary disease; *CAP* community-acquired pneumonia

The number of hospitalisations among patients 75 years old and younger increased 1.6-fold from 2006 to 2015, whereas hospitalisations among patients aged 85–89 and ≥ 90 years of age increased by 2.5- and 2.1-fold, respectively. CAP was the predominant aetiology of ARI hospitalisation. The distribution of bacterial, viral and unspecified aetiology CAP cases did not significantly differ over the 10 years. Both CAP and AECOPD hospitalisations significantly increased for all age groups during the study period (Fig. 3). The most important increases in CAP or AECOPD hospitalisations were both observed in patients aged 85 years and older.

**Fig. 4:**
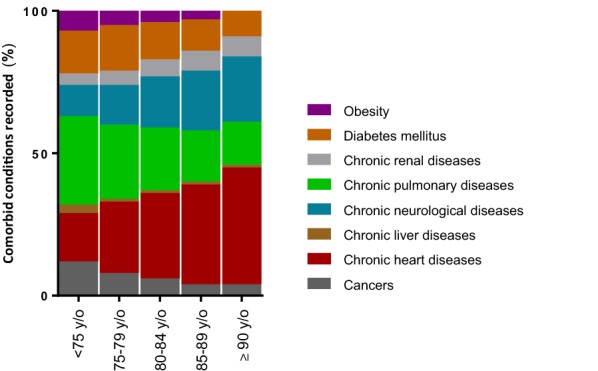
Frequency of comorbid conditions by age class. Frequency of comorbid conditions are represented for each age class. We calculated the percentage of each code on the total number of coded comorbidities recorded. We plotted the result using stacked bar graphs for each age class, with the total bar height representing 100% of comorbidity codes

The mean annual incidences of ARI were calculated for each age class throughout the study period using the annual total regional population census. The annual incidences of hospitalisation for respiratory infection clearly increased among older age groups (*p* = 0.003) and were 0.2 ± 0.03%, 1.2 ± 0.2%, 1.9 ± 0.3%, 3.2 ± 0.4% and 5.0 ± 0.7% for patients ages < 75, 75–79, 80–84, 85–89 and 90 years or older, respectively. Within the age groups, we observed changes in the coded comorbid conditions (Fig. 4). The most obvious shifts were a decrease in chronic pulmonary diseases and an increase in chronic heart diseases in the oldest patients.

### Hospitalisations in ICU and mortality

**Fig. 5:**
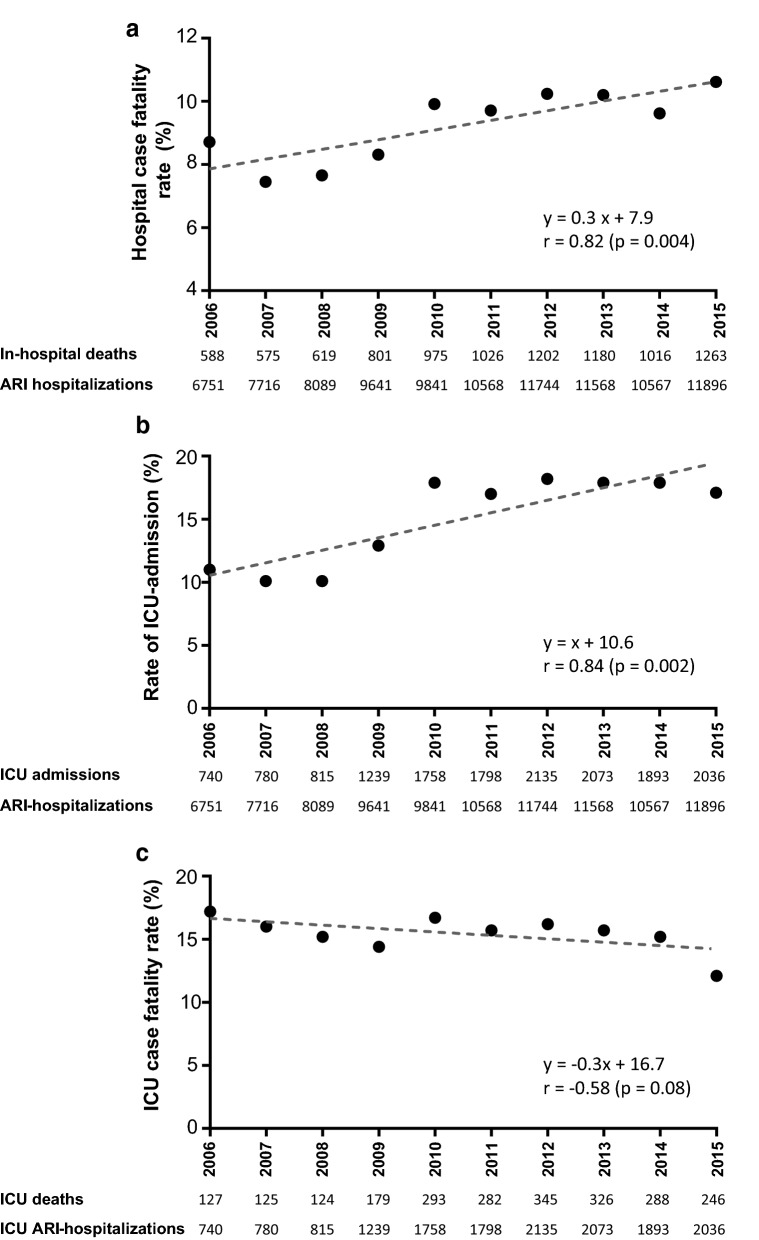
Annual trends for ARI, 2006–2015: in-hospital mortality (**a**), ICU admissions (**b**) and ICU mortality (**c**). *ARI* acute respiratory infections, *ICU* intensive care unit

As the number of ARI hospitalisations increased, so did the number of in-hospital deaths attributable to ARI: the number of in-hospital deaths increased from 588 in 2006 to 1263 in 2015. The increasing number of deaths was not only driven by the observed increase of in-hospital stays; the case fatality also significantly increased, from 8.7% in 2006 to 10.6% in 2015 (*r* = 0.82, *p* = 0.004), representing a 21.8% increase in the in-hospital mortality rate during the 10-year study period (Fig. 5a).

Among hospitalised patients with ARI, 15,267 were admitted to the ICU. The annual number of ICU hospitalisations steadily increased from 740 in 2006 to 2135 in 2012 and then remained relatively constant from 2012 to 2015, averaging 2034 admissions per year. Overall, ICU admission for ARI increased 2.7-fold from 2006 to 2015 (*r* = 0.91, *p* = 0.0002). Notably, the increase in ICU admissions was not only due to the increasing number of patients with ARI but was also because they were admitted to the ICU more often: 11.0% of ARI hospitalisations were admitted to the ICU in 2006 versus 17.1% in 2015 (*r* = 0.84, *p* = 0.002; Fig. 5b). There was an increase in the number of ICU admissions for all age groups, but the increase was highest for patients aged 85–89 and ≥ 90 years old: the number of ICU admissions among 85–89-year-old patients increased 3.3-fold, and the number of admissions among patients ≥ 90 years old increased 5.8-fold (Table 1, Additional file 1: Table S1B). Furthermore, the proportion of ARI patients admitted to the ICU who were elderly also increased over time: in 2006, 11.3% of ARI patients admitted to the ICU were aged 85 years or older compared with 16.4% in 2015 (*p* < 0.0001). The rate of ICU admissions significantly increased for all age groups during the study period. The increase in ICU hospitalisation rate was not associated with a significant change in ICU mortality (*r* = − 0.58, *p* = 0.08, Fig. 5c, Additional file 2: Figure S1), except for those ≥ 90 years old. ICU mortality for patients with ARI was 19.7 ± 3.0%, 24.0 ± 3.6% and 25.0 ± 4.0% for the patients aged 75–79, 80–84 and 85–89 years, respectively, throughout the study period. ICU mortality for patients ≥ 90 years old significantly dropped from 40.9% in 2006 to 22.3% in 2015 (*p* = 0.03).

**Fig. 6:**
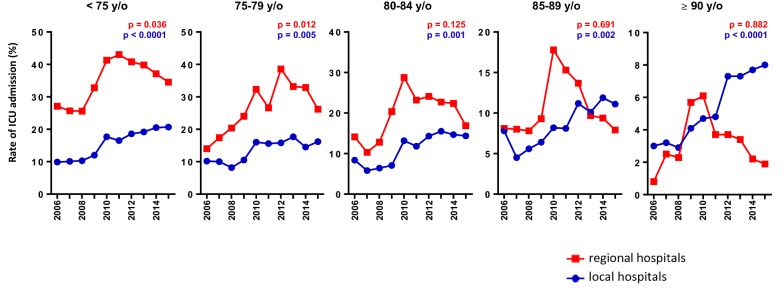
Ten-year trends in rate of ICU admission for ARI by age class according to healthcare structures. The rate of ICU hospitalisation refers to percentage of ICU hospitalisations among all hospitalisations for ARI. *ARI* acute respiratory infections, *ICU* intensive care unit

The overall rate of ICU admissions masked a certain degree of heterogeneity when comparing regional hospitals with local hospitals (Fig. 6). During the study period, there were 5923 ICU admissions for ARI in regional hospitals and 9344 ICU admissions for ARI in local hospitals. The increase in the rate of ICU admissions was observed in all the age classes in the local hospitals, including patients over 85 years of age. In the regional hospitals, there was an overall increase in the rate of ICU admission for individuals < 75 and 75–79 y/o but no significant change for those above 80 years of age over the 10-year period. We also observed a peak in ICU admission for all age groups in 2010 in these two regional hospitals.

## Discussion

This study of more than 3.8 million adult hospital stays in a French region provides a contemporary view of major epidemiological changes occurring over the last decade for patients hospitalised for ARI. We observed a rise in the number of ARI hospitalisations despite a statistically significant decline in the number of total hospitalisations (all indications combined) over the period. This substantial increase in the number of hospitalisations attributed to respiratory infections, together with an increasing demand for ICU services, led to a striking increase in the overall utilisation of ICU resources: 2038 ICU stays were recorded with ARI as the main diagnosis in 2015, compared with only 740 stays 10 years earlier (for a region of 2.5 million inhabitants). The hospitalisation of elderly patients in critical care services steadily increased for all age groups, but was greater for patients over 85 years old. Notably, ICU admissions for nonagenarians increased 5.8-fold during the decade with a marked decrease in mortality.

Respiratory infections occur mostly in elderly patients who are highly susceptible for reasons that are still poorly understood. Here, we confirmed an age-related increase in incidence, which reached 5% per year for patients over 90 years old. We did not define a cut-off value for “old” or “elderly”, because the demographic transition in Western countries is rapidly making previous definitions obsolete. Thus, the definition of an “elderly patient” is variable, and accurate comparisons between various studies are difficult. The age thresholds used in the literature to define a patient as “elderly” vary from 60 to 80 years [18–21] and from 80 to 90 for “very elderly” [11, 22, 23]. Indeed, the criteria to define an “elderly” patient are in constant flux and we preferred to use age groups instead.

Together with previous reports [22, 24], our study further demonstrates how ARI in the elderly are increasingly becoming a major public health issue. This raises important questions for future work. An important one is whether this growth in ICU hospitalisations for elderly patients with ARI is necessary to meet the demands of an expanding population requiring intensive care or whether ICU beds are being oversupplied and filled with patients who might be cared for in less-intense settings at lower cost with similar outcomes [25]. SAPS II scores and the rate of patients requiring invasive mechanical ventilation or vasopressor treatment were constant over the study period. Furthermore, the increase in ARI hospitalisations was not associated with a decrease in ICU length of stay or overall ICU case fatality rate (Fig. 5c). These results support the hypothesis that ICU admissions were driven by a real expansion of critically ill patients.

Above the individual consideration lies the societal question: how will funding agencies, policy makers and ICU caregivers face this increasing demand? Hospital stays for ARI have almost doubled over the last decade in the studied region, with a 2.7-fold increase for those in the ICU. Valley et al. [26] reported that admission to the ICU for pneumonia patients older than 64 years was associated with reduced 30-day mortality with no significant cost increase. This finding may be now assessed and confirmed for patients 85 y/o or older, for whom we observed the most important changing trends in ICU hospitalisation for ARI. Finally, the observed heterogeneity regarding triage for elderly patients with ARI highlights the need to define clear criteria for ICU admission.

Our study should be interpreted in the context of several limitations. First, the use of administrative hospital databases introduced an inherent bias that should be taken into consideration. Strengths and limitations of using healthcare databases for epidemiological purposes have already been extensively discussed [27–31]. Briefly, the PMSI (the national hospital healthcare database used here) was initially designed for billing purposes but now appears to be a powerful tool for epidemiological surveillance. However, one has to keep in mind that observed changes in disease patterns could be biased by variations in coding practices due to financial incentives for obtaining higher reimbursement rates [32]. Hence, inter-rater reliability could represent an important limitation, particularly when a single code is used. To overcome this issue, we built our case definition using an algorithm based on different diagnosis codes and their positions, and taking the variability of the coding practices into consideration. The use of coding algorithms has been validated in numerous medical fields, especially in infectious diseases. We and others have previously demonstrated that the use of an appropriate algorithm provides a high positive predictive value for a case definition and an excellent negative predictive value [30, 33]. Second, some concerning issues are not recorded in this database. For instance, increasing pollution over the past decade influences respiratory admissions; our study did not allow for testing correlations between pollution indexes and the increase in ARI hospitalisations. Similarly, some risk factors (i.e. smoking status) or protective factors (i.e. vaccination) could not be reliably studied. Third, this study population is representative of only one country, so the results may not be generalisable around the globe. Overall, the use of administrative hospital databases has strengths and weaknesses. But rather than opposing studies based on administrative data with studies based on clinical databases, future challenges will probably be to implement novel complementary strategies combining the advantages of both approaches. The major interest of this database is the exhaustive record of all patients hospitalised in the studied region during the 10-year period without initial selection bias, giving reliable information on care in real life. This could be considered a potent strategy to provide an early warning of the rise of hospitalisations for ARI. Results could be completed by more granular analyses focusing on clinical data to further decipher and explain these observations.

In conclusion, we observed a substantial increase in ARI diagnoses associated with hospitalisation between 2006 and 2015 with a growing demand for critical care services. Both the absolute number and the percentage of ICU admissions that were elderly increased over the last decade, driving an overall 2.7-fold increase in the number of ICU stays for ARI. Care delivered in the ICU contributes significantly to the expansion of healthcare spending. This work should guide physicians and healthcare administrators in their approach to policies concerning ICU admission and organisation, especially for the elderly.

## Additional files

**Additional file 1: Table S1.** Number of hospitalisation stays in the healthcare structures of the *Centre*-*Val de Loire* region (2.5 million inhabitants, France) attributable to acute respiratory infection (ARI) and according to age classes. Percentage refers to the proportion of the age class among all the patients hospitalised on the same year.

**Additional file 2: Figure S1.** Trends in ICU hospitalisations and mortality in the ICU by age class for ARI. The percentage of ICU hospitalisations refers to the rate of ICU hospitalisation for ARI among all hospitalisations for ARI. The ICU case fatality rate refers to the death rate among ICU-hospitalised patients for ARI. Four age classes are represented: patients from 75 to 79 (A), 80 to 84 (B), 85 to 89 (C) and 90 y/o or older (D). ARI: acute respiratory infections, ICU: intensive care unit.

## Abbreviations

AECOPD: acute exacerbation of chronic obstructive pulmonary disease
ARI: acute respiratory infections
CAP: community-acquired pneumonia
CVL: Centre-Val de Loire
HDD: Hospital Discharge Database
ICD-10: International Classification of Diseases, Tenth Revision
ICU: intensive care unit
y-o: years old
PMSI: *Programme de Médicalisation des Systèmes d’Information*

## References

[1] United Nations Population Division|Department of Economic and Social Affairs. http://www.un.org/en/development/desa/population/publications/ (2017). Accessed 23 Mar 2018.

[2] Kontis V, Bennett JE, Mathers CD, Li G, Foreman K, Ezzati M (2017). Future life expectancy in 35 industrialised countries: projections with a Bayesian model ensemble. Lancet Lond Engl..

[3] World Population Ageing 2013. http://www.un.org/en/development/desa/population/publications/pdf/ageing/WorldPopulationAgeing2013.pdf (2017). Accessed 23 Mar 2018.

[4] Bagshaw SM, Webb SAR, Delaney A, George C, Pilcher D, Hart GK (2009). Very old patients admitted to intensive care in Australia and New Zealand: a multi-centre cohort analysis. Crit Care Lond Engl..

[5] Reinikainen M, Uusaro A, Niskanen M, Ruokonen E (2007). Intensive care of the elderly in Finland. Acta Anaesthesiol Scand.

[6] Roch A, Wiramus S, Pauly V, Forel J-M, Guervilly C, Gainnier M (2011). Long-term outcome in medical patients aged 80 or over following admission to an intensive care unit. Crit Care Lond Engl..

[7] Tabah A, Philippart F, Timsit JF, Willems V, Français A, Leplège A (2010). Quality of life in patients aged 80 or over after ICU discharge. Crit Care Lond Engl..

[8] Pavoni V, Gianesello L, Paparella L, Buoninsegni LT, Mori E, Gori G (2012). Outcome and quality of life of elderly critically ill patients: an Italian prospective observational study. Arch Gerontol Geriatr.

[9] Andersen FH, Kvåle R (2012). Do elderly intensive care unit patients receive less intensive care treatment and have higher mortality?. Acta Anaesthesiol Scand.

[10] Puchades R, González B, Contreras M, Gullón A, de Miguel R, Martín D (2015). Cardiovascular profile in critically ill elderly medical patients: prevalence, mortality and length of stay. Eur J Intern Med..

[11] Karakus A, Haas LEM, Brinkman S, de Lange DW, de Keizer NF (2017). Trends in short-term and 1-year mortality in very elderly intensive care patients in the Netherlands: a retrospective study from 2008 to 2014. Intensive Care Med..

[12] Osler W, McCrae T. The principles and practice of medicine. New York, London, D. Appleton and company; 1920. http://archive.org/details/principlesandpr00mccrgoog (2017).

[13] Johnstone J, Marrie TJ, Eurich DT, Majumdar SR (2007). Effect of pneumococcal vaccination in hospitalized adults with community-acquired pneumonia. Arch Intern Med.

[14] Charlson ME, Pompei P, Ales KL, MacKenzie CR (1987). A new method of classifying prognostic comorbidity in longitudinal studies: development and validation. J Chronic Dis..

[15] Elixhauser A, Steiner C, Harris DR, Coffey RM (1998). Comorbidity measures for use with administrative data. Med Care.

[16] Quan H, Sundararajan V, Halfon P, Fong A, Burnand B, Luthi J-C (2005). Coding algorithms for defining comorbidities in ICD-9-CM and ICD-10 administrative data. Med Care.

[17] Estimation de la population au 1 er janvier 2016|Insee. https://www.insee.fr/fr/statistiques/1893198 (2017). Accessed 23 Mar 2018.

[18] de Rooij SE, Abu-Hanna A, Levi M, de Jonge E (2005). Factors that predict outcome of intensive care treatment in very elderly patients: a review. Crit Care Lond Engl..

[19] Boumendil A, Somme D, Garrouste-Orgeas M, Guidet B (2007). Should elderly patients be admitted to the intensive care unit?. Intensive Care Med.

[20] Peigne V, Somme D, Guérot E, Lenain E, Chatellier G, Fagon J-Y (2016). Treatment intensity, age and outcome in medical ICU patients: results of a French administrative database. Ann Intensive Care..

[21] Guidet B, Leblanc G, Simon T, Woimant M, Quenot J-P, Ganansia O (2017). Effect of systematic intensive care unit triage on long-term mortality among critically ill elderly patients in France: a randomized clinical trial. JAMA..

[22] Haas LEM, Karakus A, Holman R, Cihangir S, Reidinga AC, de Keizer NF (2015). Trends in hospital and intensive care admissions in the Netherlands attributable to the very elderly in an ageing population. Crit Care Lond Engl..

[23] Garrouste-Orgeas M, Ruckly S, Grégoire C, Dumesnil A-S, Pommier C, Jamali S (2016). Treatment intensity and outcome of nonagenarians selected for admission in ICUs: a multicenter study of the Outcomerea Research Group. Ann Intensive Care..

[24] Sjoding MW, Prescott HC, Wunsch H, Iwashyna TJ, Cooke CR (2016). Longitudinal changes in ICU admissions among elderly patients in the United States. Crit Care Med.

[25] Angus DC (2017). Admitting elderly patients to the intensive care unit—is it the right decision?. JAMA..

[26] Valley TS, Sjoding MW, Ryan AM, Iwashyna TJ, Cooke CR (2015). Association of intensive care unit admission with mortality among older patients with pneumonia. JAMA.

[27] Jouan Y, Grammatico-Guillon L, Espitalier F, Cazals X, François P, Guillon A (2015). Long-term outcome of severe herpes simplex encephalitis: a population-based observational study. Crit Care Lond Engl..

[28] Sunder S, Grammatico-Guillon L, Baron S, Gaborit C, Bernard-Brunet A, Garot D (2015). Clinical and economic outcomes of infective endocarditis. Infect Dis Lond Engl..

[29] Grammatico-Guillon L, Baron S, Gettner S, Lecuyer A-I, Gaborit C, Rosset P (2012). Bone and joint infections in hospitalized patients in France, 2008: clinical and economic outcomes. J Hosp Infect.

[30] Grammatico-Guillon L, Baron S, Gaborit C, Rusch E, Astagneau P (2014). Quality assessment of hospital discharge database for routine surveillance of hip and knee arthroplasty-related infections. Infect Control Hosp Epidemiol.

[31] Grammatico-Guillon L, Baron S, Rosset P, Gaborit C, Bernard L, Rusch E (2015). Surgical site infection after primary hip and knee arthroplasty: a cohort study using a hospital database. Infect Control Hosp Epidemiol.

[32] Rhee C, Gohil S, Klompas M (2014). Regulatory mandates for sepsis care—reasons for caution. N Engl J Med.

[33] Guillon A, Aymeric S, Gaudy-Graffin C, Sonke J, Deblonde MP, Capsec J (2017). Impact on the medical decision-making process of multiplex PCR assay for respiratory pathogens. Epidemiol Infect.

