# Supplementary material for: Ten-year trends in intensive care admissions for respiratory infections in the elderly
Source: Ann Intensive Care. 2018 Aug 15;8:84. doi: 10.1186/s13613-018-0430-6 (PMC6093821; doi:10.1186/s13613-018-0430-6)
Supplement: Supplementary file 1 — Additional file 1: Table S1. Number of hospitalisation stays in the healthcare structures of the Centre-Val de Loire region (2.5 million inhabitants, France) attributable to acute respiratory infection (ARI) and according to age classes. Percentage refers to the proportion of the age class among all the patients hospitalised on the same year. [file 13613_2018_430_MOESM1_ESM.docx]

**Additional file 2: Table S1**. **Number of hospitalization stays in the healthcare structures of the *Centre-Val de Loire* region (2.5 million inhabitants, France) attributable to acute respiratory infection (ARI) and according to age-classes.** Percentage refers to the proportion of the age-class among all the patients hospitalized on the same year.

**A. All hospitalization stays attributable to ARI**

| **Year** | **All ages** | **<75 (n,%)** | **75-79 (n,%)** | **80-84 (n,%)** | **85-89 (n,%)** | **≥90 (n,%)** |
| --- | --- | --- | --- | --- | --- | --- |
| 2006 | 6751 | 3000 (44,4) | 953 (14,1) | 1088 (16,2) | 785 (11,6) | 925 (13,7) |
| 2007 | 7716 | 3326 (43,1) | 1016 (13,2) | 1340 (17,4) | 1103 (14,2) | 931 (12,1) |
| 2008 | 8089 | 3387 (41,9) | 1096 (13,5) | 1348 (16,7) | 1293 (16,0) | 965 (11,9) |
| 2009 | 9641 | 4276 (44,4) | 1191 (12,4) | 1546 (16,0) | 1570 (16,2) | 1058 (11,0) |
| 2010 | 9841 | 4202 (42,7) | 1303 (13,2) | 1596 (16,3) | 1633 (16,6) | 1107 (11,2) |
| 2011 | 10568 | 4625 (43,8) | 1320 (12,5) | 1610 (15,2) | 1731 (16,4) | 1282 (12,1) |
| 2012 | 11744 | 4754 (40,5) | 1506 (12,8) | 1874 (16,0) | 2006 (17,1) | 1604 (13,6) |
| 2013 | 11568 | 4652 (40,2) | 1429 (12,4) | 1831 (15,8) | 2022 (17,5) | 1634 (14,1) |
| 2014 | 10567 | 4483 (42,4) | 1196 (11,3) | 1665 (15,8) | 1716 (16,2) | 1507 (14,3) |
| 2015 | 11896 | 4876 (41,0) | 1349 (11,3) | 1802 (15,2) | 1962 (16,5) | 1907 (16,0) |

**B. ICU hospitalization stays attributable to ARI**

| **Year** | **All ages** | **<75 (n,%)** | **75-79 (n,%)** | **80-84 (n,%)** | **85-89 (n,%)** | **≥90 (n,%)** |
| --- | --- | --- | --- | --- | --- | --- |
| 2006 | 740 | 442 (59,7) | 107 (14,5) | 107 (14,5) | 62 (8,3) | 22 (3,0) |
| 2007 | 780 | 482 (61,7) | 119 (15,3) | 92 (11,8) | 59 (7,5) | 28 (3,7) |
| 2008 | 815 | 484 (59,4) | 119 (14,6) | 106 (13,0) | 79 (9,7) | 27 (3,3) |
| 2009 | 1239 | 760 (61,3) | 163 (13,2) | 157 (12,7) | 112 (9,0) | 47 (3,8) |
| 2010 | 1758 | 1013 (57,6) | 257 (14,6) | 263 (15,0) | 169 (9,6) | 56 (3,2) |
| 2011 | 1798 | 1102 (61,3) | 243 (13,6) | 227 (12,6) | 168 (9,3) | 58 (3,2) |
| 2012 | 2135 | 1168 (54,7) | 321 (15,0) | 307 (14,4) | 235 (11,0) | 104 (4,9) |
| 2013 | 2073 | 1149 (55,4) | 305 (14,7) | 311 (15,0) | 203 (9,8) | 105 (5,1) |
| 2014 | 1893 | 1111 (58,7) | 218 (11,5) | 270 (14,3) | 195 (10,3) | 99 (5,2) |
| 2015 | 2036 | 1186 (58,2) | 248 (12,2) | 268 (13,2) | 207 (10,2) | 127 (6,2) |
