# Supplementary material for: Ten-year trends in intensive care admissions for respiratory infections in the elderly
Source: Ann Intensive Care. 2018 Aug 15;8:84. doi: 10.1186/s13613-018-0430-6 (PMC6093821; doi:10.1186/s13613-018-0430-6)
Supplement: Supplementary file 2 — Additional file 2: Figure S1. Trends in ICU hospitalisations and mortality in the ICU by age class for ARI. The percentage of ICU hospitalisations refers to the rate of ICU hospitalisation for ARI among all hospitalisations for ARI. The ICU case fatality rate refers to the death rate among ICU-hospitalised patients for ARI. Four age classes are represented: patients from 75 to 79 (A), 80 to 84 (B), 85 to 89 (C) and 90 y/o or older (D). ARI: acute respiratory infections, ICU: intensive care unit. [file 13613_2018_430_MOESM2_ESM.pptx]

## Slide 1
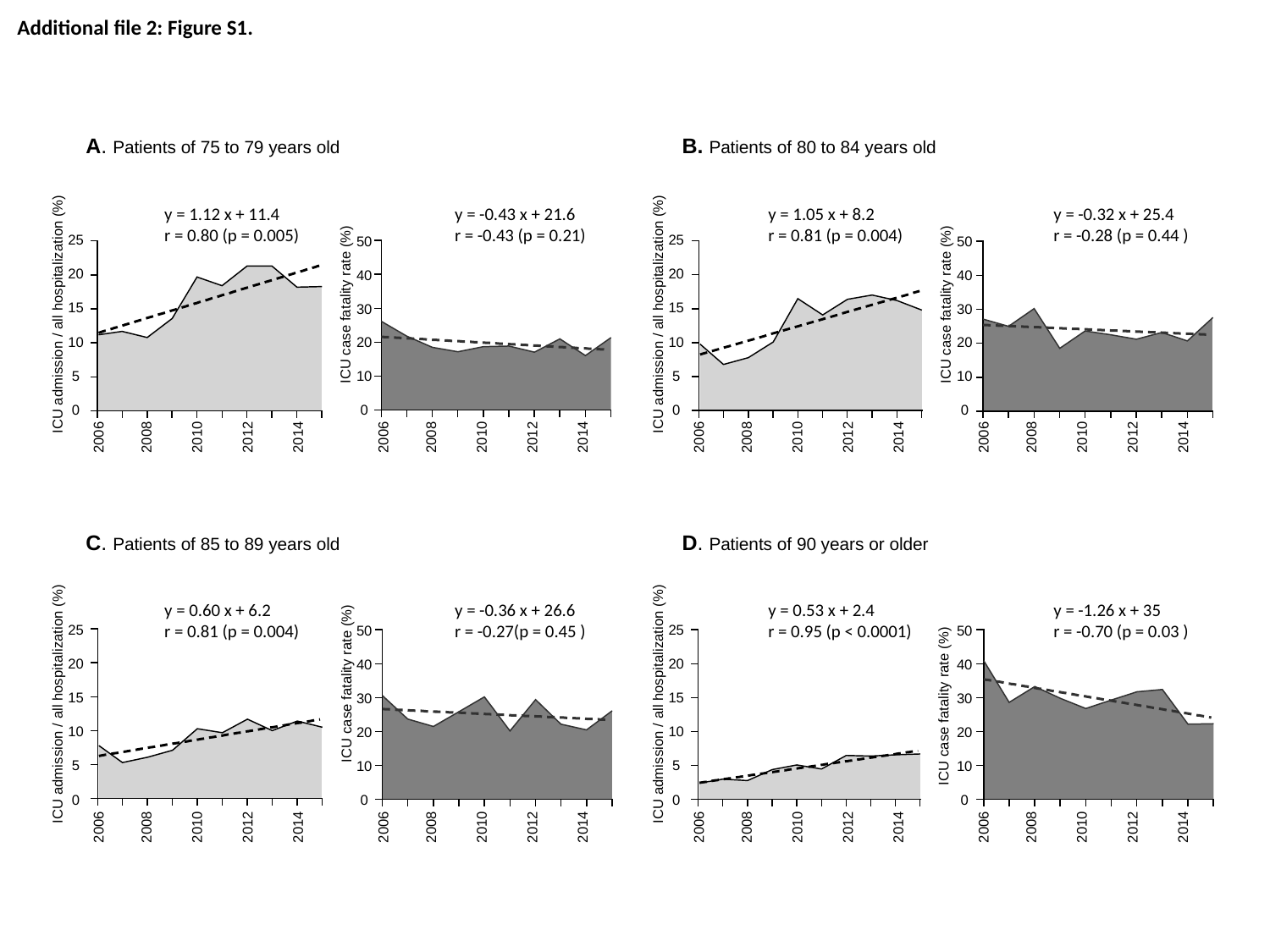

Additional file 2: Figure S1.
A. Patients of 75 to 79 years old
B. Patients of 80 to 84 years old
25
50
20
40
ICU case fatality rate (%)
15
30
ICU admission / all hospitalization (%)
10
20
5
10
0
0
2006
2008
2010
2012
2014
2006
2008
2010
2012
2014
25
50
20
40
ICU case fatality rate (%)
15
30
ICU admission / all hospitalization (%)
10
20
5
10
0
0
2006
2008
2010
2012
2014
2006
2008
2010
2012
2014
y = 1.12 x + 11.4
r = 0.80 (p = 0.005)
y = -0.43 x + 21.6
r = -0.43 (p = 0.21)
y = 1.05 x + 8.2
r = 0.81 (p = 0.004)
y = -0.32 x + 25.4
r = -0.28 (p = 0.44 )
C. Patients of 85 to 89 years old
D. Patients of 90 years or older
25
50
20
40
ICU case fatality rate (%)
15
30
ICU admission / all hospitalization (%)
10
20
5
10
0
0
2006
2008
2010
2012
2014
2006
2008
2010
2012
2014
25
50
20
40
15
30
ICU admission / all hospitalization (%)
ICU case fatality rate (%)
10
20
5
10
0
0
2006
2008
2010
2012
2014
2006
2008
2010
2012
2014
y = 0.60 x + 6.2
r = 0.81 (p = 0.004)
y = -0.36 x + 26.6
r = -0.27(p = 0.45 )
y = 0.53 x + 2.4
r = 0.95 (p < 0.0001)
y = -1.26 x + 35
r = -0.70 (p = 0.03 )
